# Causal modeling in a multi-omic setting: insights from GAW20

**DOI:** 10.1186/s12863-018-0645-4

**Published:** 2018-09-17

**Authors:** Jonathan Auerbach, Richard Howey, Lai Jiang, Anne Justice, Liming Li, Karim Oualkacha, Sergi Sayols-Baixeras, Stella W. Aslibekyan

**Affiliations:** 1 Department of Statistics, Columbia University, 1255 Amsterdam Ave, New York, NY 10027 USA; 2 Institute of Genetic Medicine, Newcastle University, Central Parkway, Newcastle-upon-Tyne, NE1 3BZ UK; 3 Department of Epidemiology, Biostatistics and Occupational Health, McGill University, 1020 Pine Avenue West, Montréal, Quebec, H3A 1A2 Canada; 4 Biomedical and Translational Informatics, Geisinger Health, 100 North Academy Ave, Danville, PA 17822 USA; 5 State Key Laboratory of Genetic Engineering, Institute of Biostatistics, School of Life Sciences, Fudan University, 2005 Songhu Road, Shanghai, 200438 China; 6 Département de Mathématiques, Université du Québec à Montréal, 2920 Chemin de la Tour, Montréal, Quebec, H3T 1 J4 Canada; 7 Cardiovascular Epidemiology and Genetics Research Group, IMIM (Hospital del Mar Medical Research Institute); Universitat Pompeu Fabra; CIBER Cardiovascular Diseases (CIBERCV), 08003 Barcelona, Catalonia Spain; 8 Department of Epidemiology, University of Alabama at Birmingham, 1665 University Blvd, RPHB 230J, Birmingham, AL 35294 USA

**Keywords:** Genomics, Epigenomics, Causal inference, Mendelian randomization, Bayesian networks, Structural equation modeling, Outliers, Variable selection methods

## Abstract

**Background:**

Increasingly available multilayered omics data on large populations has opened exciting analytic opportunities and posed unique challenges to robust estimation of causal effects in the setting of complex disease phenotypes. The GAW20 Causal Modeling Working Group has applied complementary approaches (eg, Mendelian randomization, structural equations modeling, Bayesian networks) to discover novel causal effects of genomic and epigenomic variation on lipid phenotypes, as well as to validate prior findings from observational studies.

**Results:**

Two Mendelian randomization studies have applied novel approaches to instrumental variable selection in methylation data, identifying bidirectional causal effects of *CPT1A* and triglycerides, as well as of *RNMT* and *C6orf42*, on high-density lipoprotein cholesterol response to fenofibrate. The *CPT1A* finding also emerged in a Bayesian network study. The Mendelian randomization studies have implemented both existing and novel steps to account for pleiotropic effects, which were independently detected in the GAW20 data via a structural equation modeling approach. Two studies estimated indirect effects of genomic variation (via DNA methylation and/or correlated phenotypes) on lipid outcomes of interest. Finally, a novel weighted R^2^ measure was proposed to complement other causal inference efforts by controlling for the influence of outlying observations.

**Conclusions:**

The GAW20 contributions illustrate the diversity of possible approaches to causal inference in the multi-omic context, highlighting the promises and assumptions of each method and the benefits of integrating both across methods and across omics layers for the most robust and comprehensive insights into disease processes.

## Background

The question of causality—and the distinction between association and causation—lies at the heart of scientific inquiry. In genomic research specifically, the principal focus is on estimating causal effects of DNA sequence variants on downstream phenotypes, ideally revealing the underlying biological mechanisms and identifying novel treatment targets along the way. At first glance, the task of causal inference in genomics seems trivial: individual genotype is inherited following Mendel’s laws of segregation and independent assortment, and stays stable after conception, obviating such concerns as reverse causation, confounding (except for population stratification [1]), or other spurious relationships with the disease phenotype. Yet most genome-wide association studies (GWAS) to date have failed to produce solid evidence of causality, and most statistically significant GWAS findings are often not replicated in other populations [2] or functionally corroborated by experimental models. The reasons for this failure to progress from association to causation are manifold, and include poor study design and analysis practices, the “winner’s curse [3],” low frequency of disease-causing alleles, inadequate representation of diverse populations, epistatic effects, gene–environment interactions, and various logistical and financial hurdles to conducting rigorous follow-up investigations. Largely because of these limitations, few genomic findings to date have resulted in translational breakthroughs.

The challenge of causal inference is even more daunting in methylome-wide association studies (MWAS), which interrogate associations between variation in DNA methylation and disease phenotypes. Although MWAS findings suffer from some of the same pitfalls as GWAS (eg the “winner’s curse”), they are additionally vulnerable to reverse causation and effects of confounding variables, as most of the epigenome is not heritable [4] and can be influenced by aging, disease, and a variety of environmental exposures [5]. Furthermore, single-nucleotide polymorphisms (SNPs) located in the vicinity of cytosine-phosphate-guanine (CpG) sites, which account for most of the methylation variance in the mammalian genome, have robust effects on the methylation status of the neighboring (*cis-*) CpG sites [6], potentially confounding the observed epigenomic associations with the phenotype.

The GAW20 provided an opportunity to infer causal relations between the genotype, longitudinal measures of DNA methylation, and metabolic phenotypes, collected in a large family study of lipid-lowering treatment with fenofibrate [7]. In this manuscript, we describe the methodological approaches taken by members of the GAW20 Causal Modeling Working Group, present the overarching themes and insights, and situate GAW20 findings within the broader context of genetic epidemiology research.

## Methods

The characteristics of the GAW20 data set, built from the Genetics of Lipid Lowering Drugs and Diet Network (GOLDN) study, are described in detail by Aslibekyan and colleagues [7]. Briefly, the data included epigenome-wide DNA methylation as ascertained by the Illumina Infinium Human Methylation 450 K array before and after a 3-week treatment with fenofibrate, 718,542 SNPs genotyped by the Affymetrix 6.0 array, phenotypes (plasma lipids and metabolic syndrome), and relevant covariates on 1105 individuals from 188 families. In addition to the real data from GOLDN participants, the GAW20 release included 200 replicates of simulated posttreatment methylation and phenotype (namely triglycerides) measurements, which were described by Province and colleagues [8].

Table 1 summarizes the tools and techniques employed by the 6 research teams participating in the GAW20 Causal Modeling Working Group discussion. All teams analyzed the real data, and Howey and associates [9] additionally tested their method on the simulated measurements with prior knowledge of the “answers” (ie, causal variants). Furthermore, both Jiang and associates [10] and Li and colleagues [11] conducted their own simulations to compare the performance of their methods to their predecessors. Taking advantage of the unique multi-omic context of GAW20, all teams integrated both SNP and CpG methylation data, except for Auerbach and associates, (Auerbach J, Hsu Y, Zhou W, Lo SH: Resistant R-squared for summarizing genetic effects. In preparation) who restricted their analysis to the DNA sequence variation. The team approaches represented a combination of agnostic (exploratory), genome-wide tests [11] and validation of prior reported associations (confirmatory) by using, for example, Mendelian randomization (MR) [10, 12], causal networks [9], structural equations modeling (SEM [13]), or a novel R^2^ measure that moderates the influence of outliers. Although most teams operated within established methodologic paradigms (eg, MR or SEM) [9, 10, 12, 13], Auerbach and associates and Li and colleagues [11] created new tools and tested their performance in the GAW20 data. On balance, the 6 teams varied considerably in their perspectives, approaches, and results, as discussed in further detail in the following sections.

**Table 1:** Summary of statistical methods used by the GAW20 Causal Modeling Group

	Auerbach (R^2^)	Howey (Bayesian networks)	Jiang (MR)	Justice (SEM)	Li (Mechanistic modeling)	Sayols-Baixeras (MR)
Adjustment for family relatedness	X		X	X	X	X
Bootstrapping		X	X			
Mendelian randomization			X			X
Principal components		X	X	X		
Causal networks		X		X		
Imputed SNPs			X			
Optimization		X	X			

*MR* Mendelian randomization, *SEM* structural equations modeling

## Results

### Motivation

The contributions from the Causal Modeling Working Group reflected considerable conceptual diversity. Broadly speaking, the teams aimed to strengthen the inferences that arise from observational studies reporting associations between DNA sequence or methylation variation and lipid phenotypes. In the case of Auerbach and associates, who only interrogated the genetic contribution to the phenotype, the team aimed to develop a novel R^2^ measure that would be robust to outliers. The remaining 5 studies also considered effects of epigenomic variation, which was the primary focus of the 2 MR studies [10, 12] that used genotype as the instrumental variable for methylation, phenotype, or both. In contrast to using genotype as a mere instrument, the studies by Li and colleagues [11] and Justice and associates [13] focused on sequence variation as the exposure, testing whether the total effect of the SNP on the phenotype also includes indirect effects mediated by neighboring CpG methylation or correlated lipid phenotypes. Finally, Howey and associates [9] sought to identify possible causal relationships between and within both omic layers and the phenotypes with the use of Bayesian networks. Overall, the GAW20 experience highlighted the utility of integrating across types of omic data to (a) aid causal inference and (b) paint a more complete and accurate picture of human lipid variation.

### Defining causality in GAW20

Historically, causality has been defined under 1 of 2 main frameworks, commonly referred to by their most distinctive features: potential outcomes [14] and directed graphs [15]. Both paradigms were represented among the 6 GAW20 research teams. The potential outcomes framework treats randomized controlled experiments as the gold standard for estimating causal relationships. Randomization avoids complications that occur when the manner in which subjects are assigned a treatment (or subject to an exposure) accounts for the differences in outcomes in addition to the treatment (or exposure) itself. That is not to say randomization is a statistical panacea; Auerbach and associates showed how causal estimates may be sensitive to selection effects even when treatments are randomized. Nevertheless, randomization eliminates many of the sources of confounding that could create spurious relationships and biased effect estimation. The MR approaches implemented by Jiang and colleagues [10] and Sayols-Baixeras and associates [12] represent an extension of this framework to quasiexperimental design via instrumental variable analysis [16].

In contrast, the directed graphs framework relies on deterministic laws of science for describing causal relationships. It follows that complete knowledge of the underlying mechanism of a phenomenon reveals any cause and effect relationships. In practice, it is often impossible to account for every possible relationship that might exist between a set of variables. Directed graphs take advantage of probability distributions and sequential events in time or space to simplify characterization of the data generating process. Li and associates [11] used mechanistic modeling to explain how genetic factors influence phenotype, taking advantage of the fact that genetic factors precede the phenotype. Use of structural equations to describe an outcome, represented in GAW20 by Justice and associates [13], has its origins in path diagrams [17]. The power of the directed graph framework lies in its ability to depict complicated relationships. In GAW20, Howey and associates [9] represented this framework with a Bayesian network, which consists of a directed acyclic graph and a set of parameters in all conditional probability distributions. Although this procedure is computationally expensive, it allows simultaneous analytic consideration of a large number of possible mechanisms.

**Fig. 1:**
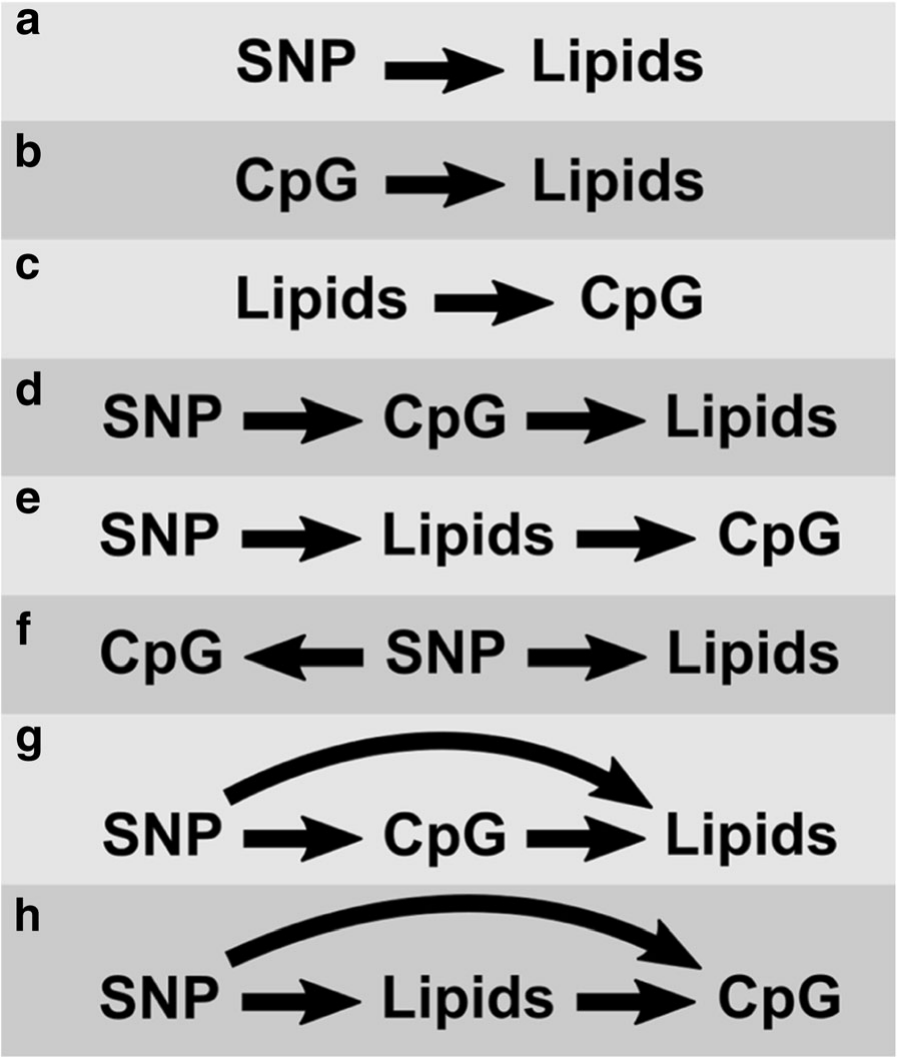
Possible (nonexhaustive) causal structures of the GAW20 data. Directed acyclic graphs illustrating several causal scenarios possible in the GAW20 data: (**a**) and (**b**) represent direct effects of DNA sequence variation and DNA methylation on lipids, respectively; (**c**) represents a direct effect of lipids on DNA methylation; (**d**) and (**e**) represent full mediation scenarios, in which the effect of DNA sequence variation on lipids/DNA methylation occurs solely through DNA methylation/lipids, respectively; (**f**) shows confounding of the DNA methylation–lipids relationship by DNA sequence variation; (**g**) and (**h**) depict partial mediation scenarios, in which DNA sequence variation affects lipids/DNA methylation both directly and through DNA methylation/lipids, respectively

Even though the interpretations of causality varied within the Causal Modeling Working Group, the teams came to the consensus definition of causal inference as the process that evaluates (and potentially rules out) competing explanations for observed associations between exposures (eg, genomic variation) and outcomes (eg, metabolic phenotypes). All analyses took place in the multi-omic setting of GAW20 data, which held several causal possibilities as summarized in Fig. 1, including confounding and reverse causation scenarios. Additionally, directed acyclic graphs (Fig. 1) can be expanded to accommodate pleiotropic effects considered by multiple GAW20 analyses [10, 12, 13]. Similarly, these graphs (Fig. 1a-h) can be modified to include repeated measurements of both methylation and phenotypic data, adding fenofibrate treatment and/or baseline lipid concentrations as potential nodes. However, even though several teams used multiple lipid measurements in their analyses, longitudinal dynamics were not a major focus of the Causal Modeling Working Group. For example, no Causal Modeling Working Group team interrogated changes in epigenetic patterns over the treatment period, likely because of the inextricable confounding between fenofibrate and batch effects on methylation measurements, described in detail elsewhere [7]. Beyond GAW20, the question of temporal variation in epigenetic effects remains similarly unexplored, but an increasing number of large-scale cohorts are currently in process of obtaining serial methylation data, promising future opportunities for adapting current causal inference methods to longitudinal epigenetics.

### Theoretical and practical challenges

#### Data

The first set of challenges for the Causal Modeling Working Group was presented by the structure of the GAW20 data set. The moderate sample size (*N* = 1105), particularly by the standards of MR analysis, hampered detection of statistically significant effects. An important step that all teams performed, but that can be often overlooked, was ensuring that the data was suitable for analysis through formatting and cleaning, such as checking Hardy-Weinberg equilibrium and/or minor allele frequencies and handling missing data. All teams adjusted for covariates (eg, age and sex) in their analyses to address bias resulting from confounding or the potential mediating effects of such variables. A special case of covariate adjustment necessary in GOLDN/GAW20 data is accounting for family relatedness, which is essential for producing valid estimates of effect in genetic studies. That was accomplished by implementing existing methods accounting for family structure [10–13], extending such methods, or not accounting for family structure while observing this limitation [9]. Potential technical artifacts in the DNA methylation data were addressed by including principal components in MWAS analyses [9, 10, 13]. Finally, as outlying observations can threaten the accuracy of estimating average effects, Auerbach and associates derived a weighted R^2^ measure that was resistant to such influences and could be used to strengthen inference from traditional statistics.

#### Analytic assumptions

The validity of causal effects estimated by all statistical methods hinges on satisfying the underlying assumptions, which are not always empirically testable. For example, MR estimators must meet the general assumptions for any instrumental variable, which include a robust association with the risk factor (testable), no common causes between the genotype and the phenotype of interest (not testable, but usually satisfied by random assortment of alleles—with the exception of population stratification), and no pleiotropic effects (ie, the genetic instrumental variable must only be associated with the phenotype of interest through the intermediate phenotype that is it meant to represent; not directly testable). To address the third assumption, Sayols-Baixeras and colleagues [12] used the widely accepted MR-Egger method [18] to rule out pleiotropy. In contrast, Jiang and associates [10] developed a novel method (constrained instrumental variables) that adaptively selects the optimal subset of instrumental variables that maximize associations with the intermediate phenotype of interest while accounting for potential pleiotropic effects. The constrained instrumental variables findings were comparable, albeit not identical, to MR-Egger and two-stage least-squares MR, identifying 2 additional causal associations as well as the 1 association detected by established methods. Meanwhile, Justice and associates [13] justified the concern about pleiotropic effects in the GAW20 data, reporting independent direct effects of *rs964184* on both triglycerides and high-density lipoprotein cholesterol, and thus indicating existence of true pleiotropy.

Similar problems exist in Bayesian network analyses [9], requiring that suitable data are included in the analysis to anchor the correct direction of the causal relationships between variables of interest. Other assumptions behind Bayesian networks include acyclic relationships between variables; multinomial distribution of discrete variables and normal distribution of continuous variables; independence between variables conditional on their parents; and no parents for SNP variables. Of those, the normality assumption is the most problematic as genomic variation is coded as 0/1/2, but is still modeled continuously to avoid problems posed by low minor allele frequencies.

The SEM analysis by Justice and colleagues [13] assumes that all data are missing at random, which is especially unlikely in longitudinal data. Justice and associates [13] found no association between missingness and any informative variable in the data set (eg, sex, age, metabolic syndrome status). However, the GAW20 data set does not contain all potentially relevant confounders that may be predictive of missingness; consequently, the missing at random assumption may not be valid.

The likelihood inference proposal for indirect estimation (LIPID) developed by Li and colleagues [11] focuses on the CpG sites that are regulated by neighboring DNA sequence variants (methylation quantitative trait loci), and also have a causal effect on the phenotype. However, current estimates indicate methylation quantitative trait loci regulation at < 40% of CpG sites [19], limiting the applicability of LIPID in studies of DNA methylation. Additionally, prior MR studies of lipids [20] demonstrated effects of the phenotype on CpG methylation rather than vice versa (ie, in the direction assumed by LIPID). Although the GAW20 findings are consistent with either direction of effect (methylation ➔ lipids and lipids ➔ methylation) [9, 12], only one direction satisfies the analytic assumption of the LIPID method.

Furthermore, both Li and colleagues [11] and Auerbach and colleagues adjusted for familial relatedness in the GAW20 data using the theoretical kinship matrix, which assumes that the founder populations are completely unrelated (which is unlikely in the context of human population history [21], particularly in close-knit communities of Utah and Minnesota that served as the study base for GOLDN/GAW20) as well as correctly specified. These issues could be obviated by estimating kinship based on SNP data rather than self-reported pedigree information [21]. The methods implemented by Li and associates [11] and Auerbach and associates also assume independence of study participants conditional on their genotype. Because environmental variables within a household are likely to be correlated, this assumption likely does not hold, and merits further investigation with a fuller data set that includes such factors as diet, lifestyle, and other potential nongenetic effects.

Finally, as all teams used linear regression models, all methods used in the Causal Modeling Working Group are based on the standard assumptions of error independence, homoscedasticity, and multivariate normality, as well as a linear relationship between the genetic/epigenetic variants and phenotypes that is unlikely to completely capture the underlying biologic complexity.

#### Subjective choices

Related to the issue of methodologic assumptions, many of the analyses used by teams required some form of subjective choices, such as weighted covariance matrix, the size of methylation probe sets [10] or SNP windows [10, 12], Bayesian network variables [9], and imputation parameters [10]. Future studies are warranted to examine the sensitivity of the proposed methods to such arbitrary initial conditions.

#### Computation

All analyses performed by the Causal Modeling Working Group faced a number of computation challenges, included but not limited to bootstrapping [9, 10], imputation [10], optimization algorithms [9], and parallelization of analyses (all teams).

## Discussion

The multilayered data environment provided by GAW20 was suitable for numerous avenues of causal inquiry, spurring vastly different approaches that unsurprisingly produced different results. One exception to that pattern was the effect of triglycerides on methylation loci in *CPT1A*, captured by both MR (in GAW20 and elsewhere [20]) and Bayesian networks, with the latter estimating the likelihood of such a causal association at 58% [9]. Notably, in addition to replicating the effect of lipids on *CPT1A* methylation, Sayols-Baixeras and associates [12] also showed that the reverse effect (ie, from methylation to triglycerides) cannot be ruled out. This is consistent with the estimate from the Bayesian network analysis, in which probability values near 50% indicate that the causal relationship is equally likely to be in either direction.

The convergence of the 2 distinct approaches on the same finding showed that the potential outcomes and the directed acyclic graph paradigms are not irreconcilable. Indeed, researchers have identified a variety of conditions necessary to bridge the 2 frameworks [15, 22, 23]. However, as a consequence of the complexity of causal inference, a variety of strategies are still useful for identifying causal relationships. In GAW20, the investigation of the *CPT1A* methylation➔ triglyceride relationship was hindered by the lack of a strong genetic instrument for the methylation loci. However, future applications of novel variable selection methods such as constrained instrumental variables [10] may be able to provide strong instruments for bidirectional MR analyses to further interrogate this epigenetic finding. Conversely, findings of causal effects of methylation in *RNMT* and *C6orf42* on high-density lipoprotein cholesterol response to fenofibrate reported by Jiang and associates [10] were subsequently bidirectionally reanalyzed to rule out reverse causality; the resulting evidence did not support a causal effect of high-density lipoprotein changes on DNA methylation. Another impediment to considering all possible causal pathways was posed by the insufficient sample size. For example, Justice and associates [13] only modeled the mediating effect of methylation on the pathway between SNP and triglyceride, because a SEM analysis examining a larger number of possible scenarios—including one where lipids act as mediators of the SNP–methylation relationship—would have been underpowered in the GAW20 data set.

Although most analyses undertook a candidate gene approach, aiming to strengthen causal inference for already known loci, Li and associates [11] used their LIPID method to identify novel genes implicated in lipid metabolism. Of 13,968 considered genes, they identified *FAT1* and *DCTN6* as having a significant effect on triglyceride phenotypes. These 2 genes were not among those examined in the other analyses of the Causal Modeling Working Group, which precludes direct comparisons with other integrative methods, such as with the structural equations models implemented by Justice and colleagues [13]. However, both *FAT1* and *DCTN6* are biologically plausible, and are annotated to lipid metabolism in the Database for Annotation, Visualization, and Integrated Discovery database [11]. Furthermore, in a rat model of in utero undernutrition followed by leptin treatment, *Dctn6* emerged as a target for “thrifty” metabolic programming [24], demonstrating its epigenetic connection to metabolic phenotypes. Given the computational efficiency of the LIPID method and its demonstrated superior statistical power compared to existing methods [11], these findings illustrate the promise of this approach for gene discovery in future integrative analyses of SNP/CpG methylation data.

## Conclusions

The experience of the GAW20 Causal Modeling Working Group illustrated several challenges and promises of causal inference in the multi-omic data environment. Employing diverse strategies to identify novel causal genes or validate prior observational findings, GAW20 contributions showed that there is no statistical “silver bullet,” as each method is predicated on its own—often not directly verifiable—assumptions. Because true causal findings are likely to be detected by more than one algorithm, future causal inference approaches can benefit from integrating multiple methods with complementary strengths and limitations. Moreover, as the availability of omics data on large population increases exponentially, it is imperative that future efforts continue exploring novel ways of leveraging all available omics layers for robust causal inference, as well as increasingly more accurate modeling of complex trait etiology.

## Abbreviations

CpG: Cytosine-phosphate-guanine
DNA: Deoxyribonucleic acid
GAW20: Genetic Analysis Workshop 20
GOLDN: Genetics of Lipid Lowering Drugs and Diet Network
GWAS: genome-wide association study
LIPID: Likelihood inference proposal for indirect estimation
MR: Mendelian randomization
MWAS: Methylome-wide association study
SEM: Structural equation modeling
SNP: Single nucleotide polymorphism
